# Dyadic Perceptions of COVID-19 Pandemic Impact on Everyday Life

**DOI:** 10.1093/geroni/igab046.3690

**Published:** 2021-12-17

**Authors:** Jennifer Margrett, Celinda Reese-Melancon, Dan Russell, Lauren Stratton, Erin Harrington, Jyoti Savla

**Affiliations:** 1 Iowa State University, Ames, Iowa, United States; 2 Oklahoma State University, Oklahoma State University, Oklahoma, United States; 3 Pennsylvania State University, University Park, Pennsylvania, United States; 4 Virginia Tech, Blacksburg, Virginia, United States

## Abstract

It is important to understand the effects of the COVID-19 pandemic not only on individuals’ daily lives, but also their close partners. Current literature suggests that the COVID-19 pandemic has impacted older adults’ lives in several ways, including the frequency of social interactions and change in various life habits (Lesbrasseur et al., 2021). Data from 42 middle-aged and older, long-term married or cohabitating dyads were collected as part of an ongoing study of everyday cognition and functioning among couples. Participant age ranged from 40-85+, and couples were partnered for 9-60+ years. During this study, COVID-19 pandemic impact was assessed using six items (1 = No change to 4 = Severe change) examining daily routines, medical and mental health access, social contacts, and pandemic and family-related stress; reports ranged from six to 19. On average, women reported significantly higher COVID-19 pandemic impact compared to men. For both partners, the greatest disruptions reported related to routines and social contacts. Further analysis examined COVID-19 pandemic impact in dyads. For eight dyads, both partners reported relatively lower COVID-19 impact (6-11), whereas for six dyads, both partners reported higher impact scores (14-19). Discussion focuses on within-dyad and between-dyad differences related to perceptions of the pandemic’s impact.

